# Supplementary material for: Unconventional linkers facilitate potent stabilized coronavirus stem antibody responses following nanoparticle vaccination
Source: bioRxiv. 2025 Nov 19:2025.11.19.689216. Preprint. [Version 1] doi: 10.1101/2025.11.19.689216 (PMC12667850; doi:10.1101/2025.11.19.689216)
Supplement: Supplement 2 [file media-2.pdf]

Unconventional linkers facilitate potent stabilized coronavirus stem antibody responses following nanoparticle vaccination

Christian K.O. Dzuvor[1,9], Sydney P. Moak[1,2,9], Lindsay R. McManus[1], Abigail E. Dzordzorme[1,3,8], Lamount R. Evanson[4], Taewoo Kim[1], Abigail Thomas[1,5], Olayimika Adeyemi[1,6], Valerie Foley[1,5], Aditi Limaye[7], Ryan P. McNamara[1], Kizzmekia S. Corbett-Helaire[1,2,10]

[1]Department of Immunology and Infectious Diseases, Harvard T.H. Chan School of Public Health; Boston, Massachusetts 02115, United States of America

[2]Howard Hughes Medical Institute; Chevy Chase, Maryland 20815, United States of America

[3]Harvard-MIT Division of Health Sciences and Technology, Institute for Medical Engineering and Science, Massachusetts Institute of Technology; Cambridge, Massachusetts 02139, United States of America

[4]Program in Virology, Harvard Medical School; Boston, Massachusetts 02115, United States of America

[5]Northeastern University; Boston, Massachusetts 02115, United States of America

[6]Delaware State University, College of Arts and Sciences; Dover, Delaware 19901, United States of America

[7]Harvard-MIT MD-PhD Program, Harvard Medical School, Boston, Massachusetts 02115, United States of America

[8]Current Affiliation: Wyss Institute for Biologically Inspired Engineering, Harvard University; Boston, Massachusetts 02215, United States of America

[9]Authors contributed equally to this study.

[10]Correspondence: kizzmekia_corbett@hsph.harvard.edu

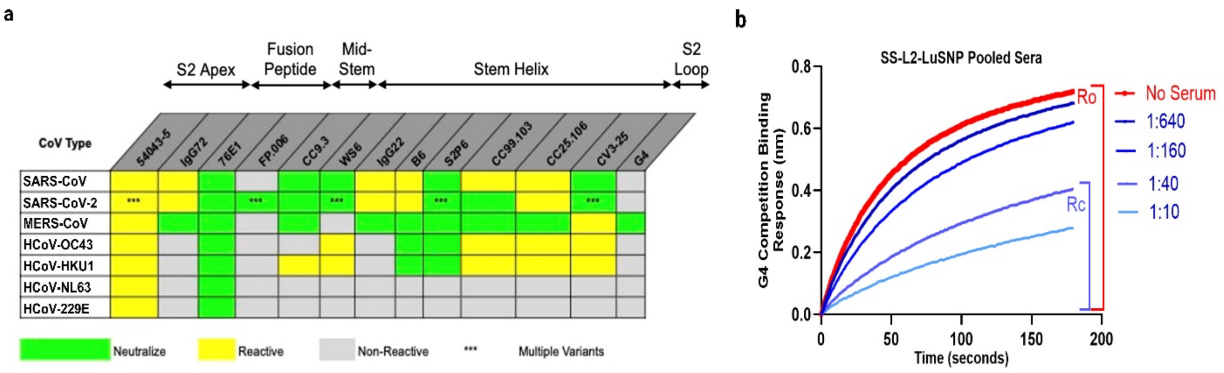

Supplementary Fig. 1: Dissecting epitope specificity of polyclonal antibody (Ab) responses by biolayer interferometry (BLI) competition assay, related to Fig. 4 (**a**) Chart of cross-reactivity across all seven human-infecting coronaviruses (CoVs). Yellow depicts reactivity, gray indicates no reactivity, and green indicates neutralization capacity. *** denotes multiple variants. (**b**) Representative binding curve for mAb G4 competing with pooled serially diluted sera from mice immunized with SS-L2-LuSNP. R_0 represents the binding signal of G4 in the absence of serum, and Rc represents the binding signal of G4 tested with sera.

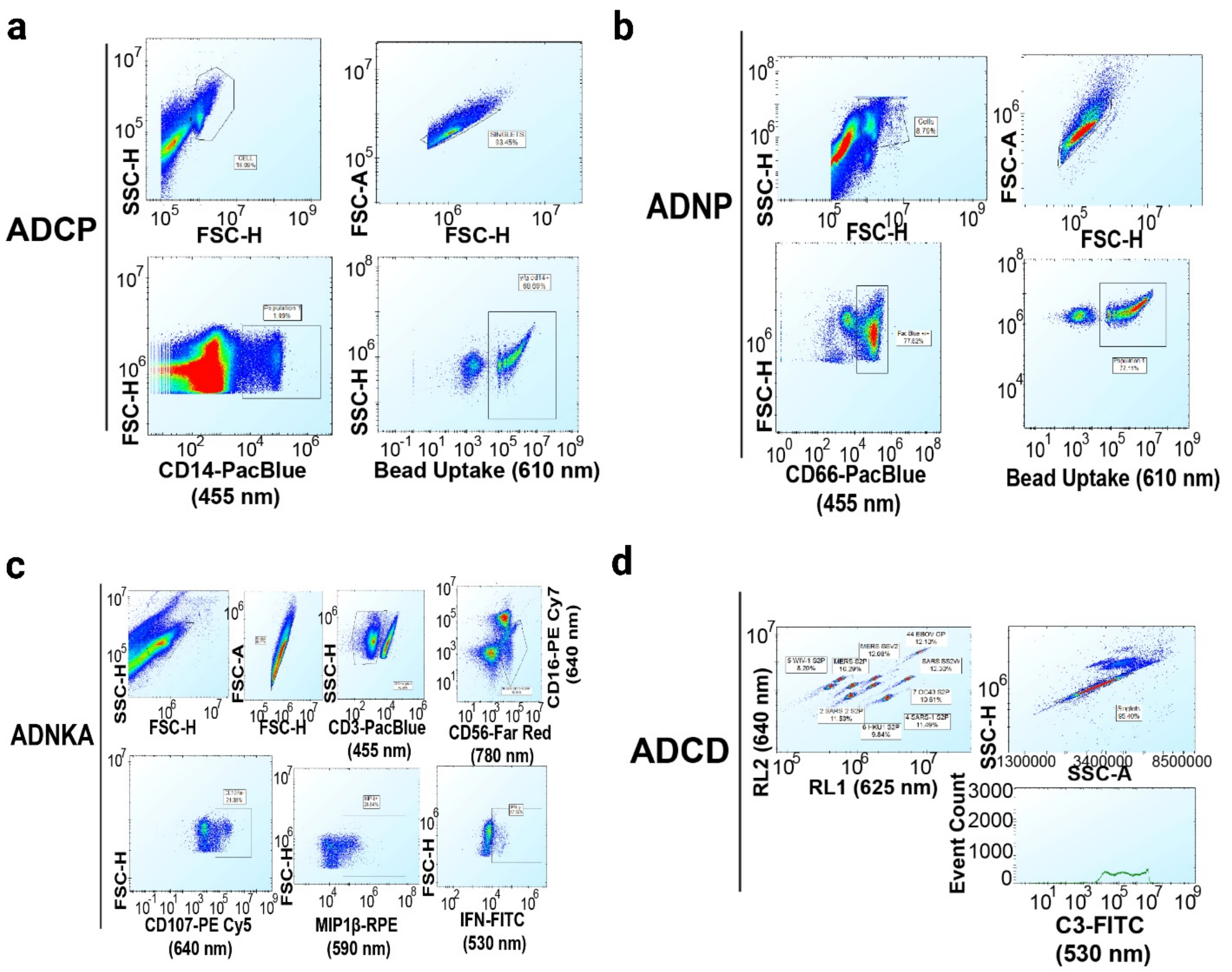

Supplementary Fig. 2: Gating strategy for Luminex-based and Ab Fc-effector function assays, related to Fig. 5. (**a**) Gating for antibody-dependent cellular phagocytosis by monocytes (ADCP) showing CD14+ monocytes and opsinophagocytosis. (**b**) Gating for antibody-dependent neutrophil phagocytosis (ADNP) showing CD66+ neutrophils and opsinophagocytosis (**c**) Gating for antibody-dependent natural killer cell activation (ADNKA) showing CD107a and MIP-1β expression on CD3-/CD56+/CD16- cells. (**d**) Gating for antibody-dependent complement deposition (ADCD) on CoV proteins on individual bead regions as measured by anti C3-FITC.

Supplementary Table 1: Conventional and *de novo* designed (unconventional) linkers.

Linker Type	Sequence	References
**Short Flexible (L0)**	GG; GS; GSG; SGG; GGGGS	1-19
**Long Flexible (L1)**	8(GS); 12(GS); 16(GS); 2(GGGGS); 4(GGS);3(GGGGS)	11,20-25
**Rigid Helical (L2)**	GGGGSAEAAAKEAAAKE EAAAKAGGGGS	This Study
**Long Flexible (L6)**	GGGSGGGGSGGGGSLSK	26

Supplementary Table 2. Cryo-EM data collection and processing parameters, related to Fig. 2e,f.

Cryo-EM Data Collection and Processing Statistics			
**EM Data Collection**	SS-L2-FerritinNP	SS-L2-LuSNP	SS-L2-153-50NP
Microscope	Talos Arctica	Talos Arctica	Talos Arctica
Software	SerialEM	SerialEM	SerialEM
Voltage(kV)	200	200	200
Detector	Gatan K3	Gatan K3	Gatan K3
Magnification(Norminal)	36,000	36,000	36,000
Electron exposure(e-/ Å^2)	50.12	60.57	57.39
Defocus range (μm)	-0.6-2.4	-0.8-2.0	0.9-2.4
Pixel size(Å)	1.1	1.1	1.1
Flux (e-/pix/ Å)	13.06	15.9	13.88
Exposure time(seconds)	4.89	4	5
Total Frames	49	46	50
Micrographs collected	1333	820	1939

3D Reconstruction Statistics			
Software	CryoSPARC v4.6.2	CryoSPARC v4.6.2	CryoSPARC v4.6.2
Symmetry imposed	O	T	I
Box size(pix)	450	450	450
Initial particle image no	1333	820	1939
Final particle image no	1300	800	1900
Map sharpening B-factor	-149	-34	-100
FSC threshold	0.143	0.143	0.143
Unmasked resolution at 0.143FSC(Å)	7.3	7.9	6.2
Masked resolution at 0.143FSC(Å)	6.2	4.1	4.4
Map resolution(Å)	6.25	4.22	4.42

Supplementary Table 3: Sequences of designed immunogens and expression constructs reported in this study.

Construct Name	Amino Acid Sequence
**Color Key**	**Secretion Signal Sequence**,MERS Stabilized S-2P, MERS-CoV Stabilized Stem(SS), Display Linker(L2), Display Linker(L6), Foldon, Streptag II, His-8-tag, FerritinNP, Lumazine Synthase (LuS)NP, I53-50A.1NT1, I53-50B.4PT1, 3C Protease Cleavage
**MERS S-2P**	**MIHSVFLLMFLLTPTES**YVDVGPDSVKSACIEVDIQQTFFDKTWPRPIDVSKADGIIYPQGRTYSNITITYQGLFPYQGDHGDMYVYSAGHATGTTPQKLFVANYSQDVKQFANGFVVRIGAAANSTGTVIISPSTSATIRKIYPAFMLGSSVGNFSDGKMGRFFNHTLVLLPDGCGTLLRAFYCILEPRSGNHCPAGNSYTSFATYHTPATDCSDGNYNRNASLNSFKEYFNLRNCTFMYTYNITEDEILEWFGITQTAQGVHLFSSRYVDLYGGNMFQFATLPVYDTIKYYSIIPHSIRSIQSDRKAWAAFYVYKLQPLTFLLDFSVDGYIRRAIDCGFNDLSQLHCSYESFDVESGVYSVSSFEAKPSGSVVEQAEGVECDFSPLLSGTPPQVYNFKRLVFTNCNYNLTKLLSLFSVNDFTCSQISPAAIASNCYSSLILDYFSYPLSMKSDLSVSSAGPISQFNYKQSFSNPTCLILATVPHNLTTITKPLKYSYINKCSRFLSDDRTEVPQLVNANQYSPCVSIVPSTVWEDGDYYRKQLSPLEGGGWLVASGSTVAMTEQLQMGFGITVQYGTDTNSVCPKLEFANDTKIASQLGNCVEYSLYGVSGRGVFQNCTAVGVRQQRFVYDAYQNLVGYYSDDGNYYCLRACVSVPVSVIYDKETKTHATLFGSVACEHISSTMSQYSRSTRSMLKRRDSTYGPLQTPVGCVLGLVNSSLFVEDCKLPLGQSLCALPDTPSTLTPASVGSVPGEMRLASIAFNHPIQVDQLNSSYFKLSIPTNFSFGVTQEYIQTTIQKVTVDCKQYVCNGFQKCEQLLREYGQFCSKINQALHGANLRQDDSVRNLFASVKSSQSSPIIPGFGGDFNLTLLEPVSISTGSRSARSAIEDLLFDKVTIADPGYMQGYDDCMQQGPASARDLICAQYVAGYKVLPPLMDVNMEAAYTSSLLGSIAGVGWTAGLSSFAAIPFAQSIFYRLNGVGITQQVLSENQKLIANKFNQALGAMQTGFTTTNEAFHKVQDAVNNNAQALSKLASELSNTFGAISASIGDIIQRLDPPEQDAQIDRLINGRLTTLNAFVAQQLVRSESAALSAQLAKDKVNECVKAQSKRSGFCGQGTHIVSFVVNAPNGLYFMHVGYYPSNHIEVVSAYGLCDAANPTNCIAPVNGYFIKTNNTRIVDEWSYTGSSFYAPEPITSLNTKYVAPQVTYQNISTNLPPPLLGNSTGIDFQDELDEFFKNVSTSIPNFGSLTQINTTLLDLTYEMLSLQQVVKALNESYIDLKELGNYTYGSGYIPEAPRDGQAYVRKDGEWVLLSTFLGRSLEVLFQGPHHHHHHHHSAWSHPQFEKGGGSGGGGSGGSAWSHPQFEK
**MERS S-2P-L2-FerritinNP**	**MIHSVFLLMFLLTPTES**YVDVGPDSVKSACIEVDIQQTFFDKTWPRPIDVSKADGIIYPQGRTYSNITITYQGLFPYQGDHGDMYVYSAGHATGTTPQKLFVANYSQDVKQFANGFVVRIGAAANSTGTVIISPSTSATIRKIYPAFMLGSSVGNFSDGKMGRFFNHTLVLLPDGCGTLLRAFYCILEPRSGNHCPAGNSYTSFATYHTPATDCSDGNYNRNASLNSFKEYFNLRNCTFMYTYNITEDEILEWFGITQTAQGVHLFSSRYVDLYGGNMFQFATLPVYDTIKYYSIIPHSIRSIQSDRKAWAAFYVYKLQPLTFLLDFSVDGYIRRAIDCGFNDLSQLHCSYESFDVESGVYSVSSFEAKPSGSVVEQAEGVECDFSPLLSGTPPQVYNFKRLVFTNCNYNLTKLLSLFSVNDFTCSQISPAAIASNCYSSLILDYFSYPLSMKSDLSVSSAGPISQFNYKQSFSNPTCLILATVPHNLTTITKPLKYSYINKCSRFLSDDRTEVPQLVNANQYSPCVSIVPSTVWEDGDYYRKQLSPLEGGGWLVASGSTVAMTEQLQMGFGITVQYGTDTNSVCPKLEFANDTKIASQLGNCVEYSLYGVSGRGVFQNCTAVGVRQQRFVYDAYQNLVGYYSDDGNYYCLRACVSVPVSVIYDKETKTHATLFGSVACEHISSTMSQYSRSTRSMLKRRDSTYGPLQTPVGCVLGLVNSSLFVEDCKLPLGQSLCALPDTPSTLTPASVGSVPGEMRLASIAFNHPIQVDQLNSSYFKLSIPTNFSFGVTQEYIQTTIQKVTVDCKQYVCNGFQKCEQLLREYGQFCSKINQALHGANLRQDDSVRNLFASVKSSQSSPIIPGFGGDFNLTLLEPV

	SISTGSRSARSAIEDLLFDKVTIADPGYMQGYDDCMQQGPASARDLICAQYVAGYKVLPPLMDVNMEAAYTSSLLGSIAGVGWTAGL<br>SSFAAIPFAQSIFYRLNGVGITQQVLSENQKLIANKFNQALGAMQTGFTTTNEAFHKVQDAVNNNAQALSKLASELSNTFGAISASIGD<br>IIQRLDPPEQDAQIDRLINGRLTTLNAFVAQQLVRSESAALSAQLAKDKVNECVKAQSKRSGFCGQGTHIVSFVVNAPNGLYFMHVG<br>YYPSNHIEVVSAYGLCDAANPTNCIAPVNGYFIKTNNTRIVDEWSYTGSSFYAPEPITSLNTKYVAPQVTYQNISTNLPPPLLGNSTGI<br>DFQDELDEFFKNVSTSIPN F GGGGS AEAAAKEAAAKEAAAKA<br>GGGGSMSSQIRQNYSTDVEAAVNSLVNLYLQASYTYLSLGFYFDRDDVALEGVSHFFRELAEEKREGYERLLKMQNQRGGRALFQ<br>DIKKPAEDEWGKTPDAMKAAMALEKKLNQALLDLHALGSARTDPHLCDFLETHFLDEEVKLIKKMGDHLTNLHRLGGPEAGLGEYLF<br>ERLTLKHD
**MERS S-2P-L6-FerritinNP**	**MIHSVFLLMFLLTPTES**YVDVGPDSVKSACIEVDIQQTFFDKTWPRPIDVSKADGIIYPQGRTYSNITITYQGLFPYQGDHGDMYVYS<br>AGHATGTTPQKLFVANYSQDVKQFANGFVVRIGAAANSTGTVIISPSTSATIRKIYPAFMLGSSVGNFSDGKMGRFFNHTLVLLPDGC<br>GTLLRAFYCILEPRSGNHCPAGNSYTSFATYHTPATDCSDGNYNRNASLNSFKEYFNLRNCTFMYTYNITEDEILEWFGITQTAQGVH<br>LFSSRYVDLYGGNMFQFATLPVYDTIKYYSIIPHSIRSIQSDRKAWAAFYVYKLQPLTFLLDFSVDGYIRRAIDCGFNDLSQLHCSYESF<br>DVESGVYSVSSFEAKPSGSVVEQAEGVECDFSPLLSGTPPQVYNFKRLVFTNCNYNLTKLLSLFSVNDFTCSQISPAAIASNCYSSLI<br>LDYFSYPLSMKSDLSVSSAGPISQFNYKQSFSNPTCLILATVPHNLTTITKPLKYSYINKCSRFLSDDRTEVPQLVNANQYSPCVSIVP<br>STVWEDGDYYRKQLSPLEGGGWLVASGSTVAMTEQLQMGFGITVQYGTDTNSVCPKLEFANDTKIASQLGNCVEYSLYGVSGRGV<br>FQNCTAVGVRQQRFVYDAYQNLVGYYSDDGNYYCLRACVSVPVSVIYDKETKTHATLFGSVACEHISSTMSQYSRSTRSMLKRRDS<br>TYGPLQTPVGCVLGLVNSSLFVEDCKLPLGQSLCALPDTPSTLTPASVGSVPGEMRLASIAFNHPIQVDQLNSSYFKLSIPTNFSFGV<br>TQEYIQTTIQKVTVDCKQYVCNGFQKCEQLLREYGQFCSKINQALHGANLRQDDSVRNLFASVKSSQSSPIIPGFGGDFNLTLLEPV<br>SISTGSRSARSAIEDLLFDKVTIADPGYMQGYDDCMQQGPASARDLICAQYVAGYKVLPPLMDVNMEAAYTSSLLGSIAGVGWTAGL<br>SSFAAIPFAQSIFYRLNGVGITQQVLSENQKLIANKFNQALGAMQTGFTTTNEAFHKVQDAVNNNAQALSKLASELSNTFGAISASIGD<br>IIQRLDPPEQDAQIDRLINGRLTTLNAFVAQQLVRSESAALSAQLAKDKVNECVKAQSKRSGFCGQGTHIVSFVVNAPNGLYFMHVG<br>YYPSNHIEVVSAYGLCDAANPTNCIAPVNGYFIKTNNTRIVDEWSYTGSSFYAPEPITSLNTKYVAPQVTYQNISTNLPPPLLGNSTGI<br>DFQDELDEFFKNVSTSIPN FGS<br>GGGSGGGGSGGGGSGLSKMSSQIRQNYSTDVEAAVNSLVNLYLQASYTYLSLGFYFDRDDVALEGVSHFFRELAEEKREGYERLL<br>KMQNQRGGRALFQDIKKPAEDEWGKTPDAMKAAMALEKKLNQALLDLHALGSARTDPHLCDFLETHFLDEEVKLIKKMGDHLTNLH<br>RLGGPEAGLGEYLFERLTLKHD
**MERS S-2P-L2-LuSNP**	**MIHSVFLLMFLLTPTES**YVDVGPDSVKSACIEVDIQQTFFDKTWPRPIDVSKADGIIYPQGRTYSNITITYQGLFPYQGDHGDMYVYS<br>AGHATGTTPQKLFVANYSQDVKQFANGFVVRIGAAANSTGTVIISPSTSATIRKIYPAFMLGSSVGNFSDGKMGRFFNHTLVLLPDGC<br>GTLLRAFYCILEPRSGNHCPAGNSYTSFATYHTPATDCSDGNYNRNASLNSFKEYFNLRNCTFMYTYNITEDEILEWFGITQTAQGVH<br>LFSSRYVDLYGGNMFQFATLPVYDTIKYYSIIPHSIRSIQSDRKAWAAFYVYKLQPLTFLLDFSVDGYIRRAIDCGFNDLSQLHCSYESF<br>DVESGVYSVSSFEAKPSGSVVEQAEGVECDFSPLLSGTPPQVYNFKRLVFTNCNYNLTKLLSLFSVNDFTCSQISPAAIASNCYSSLI<br>LDYFSYPLSMKSDLSVSSAGPISQFNYKQSFSNPTCLILATVPHNLTTITKPLKYSYINKCSRFLSDDRTEVPQLVNANQYSPCVSIVP<br>STVWEDGDYYRKQLSPLEGGGWLVASGSTVAMTEQLQMGFGITVQYGTDTNSVCPKLEFANDTKIASQLGNCVEYSLYGVSGRGV<br>FQNCTAVGVRQQRFVYDAYQNLVGYYSDDGNYYCLRACVSVPVSVIYDKETKTHATLFGSVACEHISSTMSQYSRSTRSMLKRRDS

	TYGPLQTPVGCVLGLVNSSLFVEDCKLPLGQSLCALPDTPSTLTPASVGSVPGEMRLASIAFNHPIQVDQLNSSYFKLSIPTNFSFGV TQEYIQTTIQKVTVDCKQYVCNGFQKCEQLLREYGQFCSKINQALHGANLRQDDSVRNLFASVKSSQSSPIIPGFGGDFNLTLLEPV SISTGSRSARSAIEDLLFDKVTIADPGYMQGYDDCMQQGPASARDLICAQYVAGYKVLPPLMDVNMEAAYTSSLLGSIAGVGWTAGL SSFAAIPFAQSIFYRLNGVGITQQVLSENQKLIANKFNQALGAMQTGFTTTNEAFHKVQDAVNNNAQALSKLASELSNTFGAISASIGD IIQRLDPPEQDAQIDRLINGRLTTLNAFVAQQLVRSESAALSAQLAKDKVNECVKAQSKRSGFCGQGTHIVSFVVNAPNGLYFMHVG YYPSNHIEVVSAYGLCDAANPTNCIAPVNGYFIKTNNTRIVDEWSYTGSSFYAPEPITSLNTKYVAPQVTYQNISTNLPPPLLGNSTGI DFQDELDEFFKNVSTSIPN FGGGGS AEAAAKEAAAKEAAAKA GGGGS<br>MQIYEGKLTAEGLRFGIVASRFNHALVDRLVEGAIDCIVRHGGREEDITLVRVPGSWEIPVAAGELARKEDIDAVIAIGVLIRGATPHFDY IASEVSKGLANLSLELRKPITFGVITADTLEQAIERAGTKHGNKGWEAALSAIEMANLFKSLR
**MERS S-2P-L6-LuSNP**	**MIHSVFLLMFLLTPTES**YVDVGPDSVKSACIEVDIQQTFFDKTWPRPIDVSKADGIIYPQGRTYSNITITYQGLFPYQGDHGDMYVYS AGHATGTTPQKLFVANYSQDVKQFANGFVVRIGAAANSTGTVIISPSTSATIRKIYPAFMLGSSVGNFSDGKMGRFFNHTLVLLPDGC GTLLRAFYCILEPRSGNHCPAGNSYTSFATYHTPATDCSDGNYNRNASLNSFKEYFNLRNCTFMYTYNITEDEILEWFGITQTAQGVH LFSSRYVDLYGGNMFQFATLPVYDTIKYYSIIPHSIRSIQSDRKAWAAFYVYKLQPLTFLLDFSVDGYIRRAIDCGFNDLSQLHCSYESF DVESGVYSVSSFEAKPSGSVVEQAEGVECDFSPLLSGTPPQVYNFKRLVFTNCNYNLTKLLSLFSVNDFTCSQISPAAIASNCYSSLI LDYFSYPLSMKSDLSVSSAGPISQFNYKQSFSNPTCLILATVPHNLTTITKPLKYSYINKCSRFLSDDRTEVPQLVNANQYSPCVSIVP STVWEDGDYYRKQLSPLEGGGWLVASGSTVAMTEQLQMGFGITVQYGTDTNSVCPKLEFANDTKIASQLGNCVEYSLYGVSGRGV FQNCTAVGVRQQRFVYDAYQNLVGYYSDDGNYYCLRACVSVPVSVIYDKETKTHATLFGSVACEHISSTMSQYSRSTRSMLKRRDS TYGPLQTPVGCVLGLVNSSLFVEDCKLPLGQSLCALPDTPSTLTPASVGSVPGEMRLASIAFNHPIQVDQLNSSYFKLSIPTNFSFGV TQEYIQTTIQKVTVDCKQYVCNGFQKCEQLLREYGQFCSKINQALHGANLRQDDSVRNLFASVKSSQSSPIIPGFGGDFNLTLLEPV SISTGSRSARSAIEDLLFDKVTIADPGYMQGYDDCMQQGPASARDLICAQYVAGYKVLPPLMDVNMEAAYTSSLLGSIAGVGWTAGL SSFAAIPFAQSIFYRLNGVGITQQVLSENQKLIANKFNQALGAMQTGFTTTNEAFHKVQDAVNNNAQALSKLASELSNTFGAISASIGD IIQRLDPPEQDAQIDRLINGRLTTLNAFVAQQLVRSESAALSAQLAKDKVNECVKAQSKRSGFCGQGTHIVSFVVNAPNGLYFMHVG YYPSNHIEVVSAYGLCDAANPTNCIAPVNGYFIKTNNTRIVDEWSYTGSSFYAPEPITSLNTKYVAPQVTYQNISTNLPPPLLGNSTGI DFQDELDEFFKNVSTSIPN FGS GGGSGGGGSGGGGSGLSK<br>MQIYEGKLTAEGLRFGIVASRFNHALVDRLVEGAIDCIVRHGGREEDITLVRVPGSWEIPVAAGELARKEDIDAVIAIGVLIRGATPHFDY IASEVSKGLANLSLELRKPITFGVITADTLEQAIERAGTKHGNKGWEAALSAIEMANLFKSLR
**SS**	**MRPTWAWWLFLVLLLALWAPARG**QAFNHPIQVDQLNSSYFKLSIPTNFSFGVTQEYIQTTIQKVCVDCKQYVCNGFQKCEQLLREY GQFCSKINQALHGCNLRQDDSVRNLFASVKSSQSSPIIPGFGGDFNLTLLEPVSISTGSRSARSAIEDLLFDKCTIADPGYMQGYDDC MQQGPASARDLICAQYVAGYCVLPPLMDVNMEAAYTSSLLGSIAGSGWTAGLSSFAAIPFAQMIFYRLNGIGITQQVLSENQKLIANK FNQALGAMQTGFTTTNEAFHKCQDAVNNNAQALSKLASELSNTFGAISASIGDIIQRLDPPEQDAQIDRLINGRLTTLNAFVAQQLVR CEEAAQSAQLAKDKVNECVKAQSKRSGFCGQGTHIVSFVVNAPYGLYFMHVGYYPSNHIEVVSAYGLCDAANPTNCIAPVNGYFIK TNNTRIVDEWSYTGSSFYAPEPITSLNTKYVAPQVTYQNISTNLPPPLLGNSTGIDFQDELDEFFKNVSTSIPNFGSLTQINTTLLDLTY EMLSLQQVVKALNESYIDLKELGNYTYGSGYIPEAPRDGQAYVRKDGEWVLLSTFLGGRLEVLFQGPGGYIPEAPRDGQAYVRKDG EWVLLSTFLGHHHHHHHHSAWSHPQFEK

**SS-L2-FerritinNP**	**MRPTWAWWLFLVLLLALWAPARG**QAFNHPIQVDQLNSSYFKLSIPTNFSFGVTQEYIQTTIQKVCVDCKQYVCNGFQKCEQLLREYGQFCSKINQALHGCNLRQDDSVRNLFASVKSSQSSPIIPGFGGDFNLTLLEPVSISTGSRSARSAIEDLLFDKCTIADPGYMQGYDDCMQQGPASARDLICAQYVAGYCVLPPLMDVNMEAAYTSSLLGSIAGSGWTAGLSSFAAIPFAQMIFYRLNGIGITQQVLSENQKLIANKFNQALGAMQTGFTTTNEAFHKCQDAVNNNAQALSKLASELSNTFGAISASIGDIIQRLDPPEQDAQIDRLINGRLTTLNAFVAQQLVRCEEAAQSAQLAKDKVNECVKAQSKRSGFCGQGTHIVSFVVNAPYGLYFMHVGYYPSNHIEVVSAYGLCDAANPTNCIAPVNGYFIKTNNTRIVDEWSYTGSSFYAPEPITSLNTKYVAPQVTYQNISTNLPPPLLGNSTGIDFQDELDEFFKNVSTSIPNFGSLTQINTTLLDLTYEMLSLQQVVKALNESYIDLKELGNYTYGSGYIPEAPRDGQAYVRKDGEWVLLSTFLG**GGGGSAEAAAKEAAAKEAAAKAGGGGS**MSSQIRQNYSTDVEAAVNSLVNLYLQASYTYLSLGFYFDRDDVALEGVSHFFRELAEEKREGYERLLKMQNQRGGRALFQDIKKPAEDEWGKTPDAMKAAMALEKKLNQALLDLHALGSARTDPHLCDFLETHFLDEEVKLIKKMGDHLTNLHRLGGPEAGLGEYLFERLTLKHD
**SS-L6-FerritinNP**	**MRPTWAWWLFLVLLLALWAPARG**QAFNHPIQVDQLNSSYFKLSIPTNFSFGVTQEYIQTTIQKVCVDCKQYVCNGFQKCEQLLREYGQFCSKINQALHGCNLRQDDSVRNLFASVKSSQSSPIIPGFGGDFNLTLLEPVSISTGSRSARSAIEDLLFDKCTIADPGYMQGYDDCMQQGPASARDLICAQYVAGYCVLPPLMDVNMEAAYTSSLLGSIAGSGWTAGLSSFAAIPFAQMIFYRLNGIGITQQVLSENQKLIANKFNQALGAMQTGFTTTNEAFHKCQDAVNNNAQALSKLASELSNTFGAISASIGDIIQRLDPPEQDAQIDRLINGRLTTLNAFVAQQLVRCEEAAQSAQLAKDKVNECVKAQSKRSGFCGQGTHIVSFVVNAPYGLYFMHVGYYPSNHIEVVSAYGLCDAANPTNCIAPVNGYFIKTNNTRIVDEWSYTGSSFYAPEPITSLNTKYVAPQVTYQNISTNLPPPLLGNSTGIDFQDELDEFFKNVSTSIPNFGSLTQINTTLLDLTYEMLSLQQVVKALNESYIDLKELGNYTYGSGYIPEAPRDGQAYVRKDGEWVLLSTFLG**GGGSGGGGSGGGGSGLSK**MSSQIRQNYSTDVEAAVNSLVNLYLQASYTYLSLGFYFDRDDVALEGVSHFFRELAEEKREGYERLLKMQNQRGGRALFQDIKKPAEDEWGKTPDAMKAAMALEKKLNQALLDLHALGSARTDPHLCDFLETHFLDEEVKLIKKMGDHLTNLHRLGGPEAGLGEYLFERLTLKHD
**SS-L2-LuSNP**	**MRPTWAWWLFLVLLLALWAPARG**QAFNHPIQVDQLNSSYFKLSIPTNFSFGVTQEYIQTTIQKVCVDCKQYVCNGFQKCEQLLREYGQFCSKINQALHGCNLRQDDSVRNLFASVKSSQSSPIIPGFGGDFNLTLLEPVSISTGSRSARSAIEDLLFDKCTIADPGYMQGYDDCMQQGPASARDLICAQYVAGYCVLPPLMDVNMEAAYTSSLLGSIAGSGWTAGLSSFAAIPFAQMIFYRLNGIGITQQVLSENQKLIANKFNQALGAMQTGFTTTNEAFHKCQDAVNNNAQALSKLASELSNTFGAISASIGDIIQRLDPPEQDAQIDRLINGRLTTLNAFVAQQLVRCEEAAQSAQLAKDKVNECVKAQSKRSGFCGQGTHIVSFVVNAPYGLYFMHVGYYPSNHIEVVSAYGLCDAANPTNCIAPVNGYFIKTNNTRIVDEWSYTGSSFYAPEPITSLNTKYVAPQVTYQNISTNLPPPLLGNSTGIDFQDELDEFFKNVSTSIPNFGSLTQINTTLLDLTYEMLSLQQVVKALNESYIDLKELGNYTYGSGYIPEAPRDGQAYVRKDGEWVLLSTFLG**GGGGSAEAAAKEAAAKEAAAKAGGGGS**MQIYEGKLTAEGLRFGIVASRFNHALVDRLVEGAIDCIVRHGGREEDITLVRVPGSWEIPVAAGELARKEDIDAVIAIGVLIRGATPHFDYIASEVSKGLANLSLELRKPITFGVITADTLEQAIERAGTKHGNKGWEAALSAIEMANLFKSLR
**SS-L6-LuSNP**	**MRPTWAWWLFLVLLLALWAPARG**QAFNHPIQVDQLNSSYFKLSIPTNFSFGVTQEYIQTTIQKVCVDCKQYVCNGFQKCEQLLREYGQFCSKINQALHGCNLRQDDSVRNLFASVKSSQSSPIIPGFGGDFNLTLLEPVSISTGSRSARSAIEDLLFDKCTIADPGYMQGYDDCMQQGPASARDLICAQYVAGYCVLPPLMDVNMEAAYTSSLLGSIAGSGWTAGLSSFAAIPFAQMIFYRLNGIGITQQVLSENQKLIANKFNQALGAMQTGFTTTNEAFHKCQDAVNNNAQALSKLASELSNTFGAISASIGDIIQRLDPPEQDAQIDRLINGRLTTLNAFVAQQLVRCEEAAQSAQLAKDKVNECVKAQSKRSGFCGQGTHIVSFVVNAPYGLYFMHVGYYPSNHIEVVSAYGLCDAANPTNCIAPVNGYFIKTNNTRIVDEWSYTGSSFYAPEPITSLNTKYVAPQVTYQNISTNLPPPLLGNSTGIDFQDELDEFFKNVSTSIPNFGSLTQINTTLLDLTY

	EMLSLQQVVKALNESYIDLKELGNYTYGSGYIPEAPRDGQAYVRKDGEWVLLSTFLG**GGGSGGGGSGGGGSGLSK**MQIYEGKLTA<br>EGLRFGIVASRFNHALVDRLVEGAIDCIVRHGGREEDITLVRVPGSWEIPVAAGELARKEDIDAVIAIGVLIRGATPHFDYIASEVSKGLA<br>NLSLELRKPITFGVITADTLEQAIERAGTKHGNKGWEAALSAIEMANLFKSLR
**SS-L2-I53-50A.1NT1**	**MRPTWAWWLFLVLLLALWAPARG**QAFNHPIQVDQLNSSYFKLSIPTNFSFGVTQEYIQTTIQKVCVDCKQYVCNGFQKCEQLLREY<br>GQFCSKINQALHGCNLRQDDSVRNLFASVKSSQSSPIIPGFGGDFNLTLLEPVSISTGSRSARSAIEDLLFDKCTIADPGYMQGYDDC<br>MQQGPASARDLICAQYVAGYCVLPPLMDVNMEAAYTSSLLGSIAGSGWTAGLSSFAAIPFAQMIFYRLNGIGITQQVLSENQKLIANK<br>FNQALGAMQTGFTTTNEAFHKCQDAVNNNAQALSKLASELSNTFGAISASIGDIIQRLDPPEQDAQIDRLINGRLTTLNAFVAQQLVR<br>CEEAAQSAQLAKDKVNECVKAQSKRSGFCGQGTHIVSFVVNAPYGLYFMHVGYYPSNHIEVVSAYGLCDAANPTNCIAPVNGYFIK<br>TNNTRIVDEWSYTGSSFYAPEPITSLNTKYVAPQVTYQNISTNLPPPLLGNSTGIDFQDELDEFFKNVSTSIPNFGSLTQINTTLLDLTY<br>EMLSLQQVVKALNESYIDLKELGNYTYGSGYIPEAPRDGQAYVRKDGEWVLLSTFLG**GGGGSAEAAAKEAAAKEAAAKAGGGGS**<br>MEELFKKHKIVAVLRANSVEEAIEKAVAVFAGGVHLIEITFTVPDADTVIKALSVLKEKGAIIGAGTVTSVEQCRKAVESGAEFIVSPHL<br>DEEISQFCKEKGVFYMPGVMTPTELVKAMKLGHDILKLFPGEVVGPEFVKAMKGPFPNVKFVPTGGVDLDNVCEWFDAGVLAVGV<br>GDALVEGDPDEVREKAKEFVEKIRGCTEGSLEWSHPQFEKGSGHHHHHHHH
**SS-L6-I53-50A.1NT1**	**MRPTWAWWLFLVLLLALWAPARG**QAFNHPIQVDQLNSSYFKLSIPTNFSFGVTQEYIQTTIQKVCVDCKQYVCNGFQKCEQLLREY<br>GQFCSKINQALHGCNLRQDDSVRNLFASVKSSQSSPIIPGFGGDFNLTLLEPVSISTGSRSARSAIEDLLFDKCTIADPGYMQGYDDC<br>MQQGPASARDLICAQYVAGYCVLPPLMDVNMEAAYTSSLLGSIAGSGWTAGLSSFAAIPFAQMIFYRLNGIGITQQVLSENQKLIANK<br>FNQALGAMQTGFTTTNEAFHKCQDAVNNNAQALSKLASELSNTFGAISASIGDIIQRLDPPEQDAQIDRLINGRLTTLNAFVAQQLVR<br>CEEAAQSAQLAKDKVNECVKAQSKRSGFCGQGTHIVSFVVNAPYGLYFMHVGYYPSNHIEVVSAYGLCDAANPTNCIAPVNGYFIK<br>TNNTRIVDEWSYTGSSFYAPEPITSLNTKYVAPQVTYQNISTNLPPPLLGNSTGIDFQDELDEFFKNVSTSIPNFGSLTQINTTLLDLTY<br>EMLSLQQVVKALNESYIDLKELGNYTYGSGYIPEAPRDGQAYVRKDGEWVLLSTFLG**GGGSGGGGSGGGGSGLSK**MEELFKKHKI<br>VAVLRANSVEEAIEKAVAVFAGGVHLIEITFTVPDADTVIKALSVLKEKGAIIGAGTVTSVEQCRKAVESGAEFIVSPHLDEEISQFCKE<br>KGVFYMPGVMTPTELVKAMKLGHDILKLFPGEVVGPEFVKAMKGPFPNVKFVPTGGVDLDNVCEWFDAGVLAVGVGDALVEGDP<br>DEVREKAKEFVEKIRGCTEGSLEWSHPQFEKGSGHHHHHHHH
**I53-50A.1NT1**	MKMEELFKKHKIVAVLRANSVEEAIEKAVAVFAGGVHLIEITFTVPDADTVIKALSVLKEKGAIIGAGTVTSVEQCRKAVESGAEFIVSP<br>HLDEEISQFCKEKGVFYMPGVMTPTELVKAMKLGHDILKLFPGEVVGPEFVKAMKGPFPNVKFVPTGGVDLDNVCEWFDAGVLAV<br>GVGDALVEG DPDEVREKAKEFVEKIRGCTEGSLEHHHHHHHH
**I53-50B.4PT1**	MNQHSHKDHETVRIAVVRARWHAEIVDACVSAFEAAMRDIGGDRFAVDVFDVPGAYEIPLHARTLAETGRYGAVLGTAFVVNGGIY<br>RHEFVASAVINGMMNVQLNTGVPVLSAVLTPHNYDKSKAHTLLFLALFAVKGMEAARACVEILAAREKIAAGSLEHHHHHHHH

References, related to Supplementary Table 1

1. Brinkkemper, M. *et al.* Co-display of diverse spike proteins on nanoparticles broadens sarbecovirus neutralizing antibody responses. *iScience* **25**, 105649-105649 (2022). https://doi.org/10.1016/j.isci.2022.105649
2. Joyce, M. G. *et al.* SARS-CoV-2 ferritin nanoparticle vaccines elicit broad SARS coronavirus immunogenicity. *Cell Reports* **37**, 110143 (2021). https://doi.org/10.1016/j.celrep.2021.110143
3. Hutchinson, G. B. *et al.* Nanoparticle display of prefusion coronavirus spike elicits S1-focused cross-reactive antibody response against diverse coronavirus subgenera. *Nature Communications* **14** (2023). https://doi.org/10.1038/s41467-023-41661-4
4. Antanasijevic, A. *et al.* Structural and functional evaluation of de novo-designed, two-component nanoparticle carriers for HIV Env trimer immunogens. *PLOS Pathogens* **16**, e1008665 (2020). https://doi.org/10.1371/journal.ppat.1008665
5. Boyoglu-Barnum, S. *et al.* Quadrivalent influenza nanoparticle vaccines induce broad protection. *Nature* **592**, 623-628 (2021). https://doi.org/10.1038/s41586-021-03365-x
6. Kraft, J. C. *et al.* Antigen- and scaffold-specific antibody responses to protein nanoparticle immunogens. *Cell Reports Medicine* **3**, 100780 (2022). https://doi.org/10.1016/j.xcrm.2022.100780
7. Kanekiyo, M. *et al.* Mosaic nanoparticle display of diverse influenza virus hemagglutinins elicits broad B cell responses. *Nature Immunology* **20**, 362-372 (2019). https://doi.org/10.1038/s41590-018-0305-x
8. Ueda, G. *et al.* (Cold Spring Harbor Laboratory, 2020).
9. Brouwer, P. J. M. *et al.* Lassa virus glycoprotein nanoparticles elicit neutralizing antibody responses and protection. *Cell Host & Microbe* **30**, 1759-1772.e1712 (2022). https://doi.org/10.1016/j.chom.2022.10.018
10. Hu, Z. *et al.* Nanoparticle vaccine based on the pre-fusion F glycoprotein of respiratory syncytial virus elicits robust protective immune responses. *Journal of Virology* **99** (2025). https://doi.org/10.1128/jvi.00903-25
11. Zhang, Y.-N. *et al.* Single-component multilayered self-assembling protein nanoparticles presenting glycan-trimmed uncleaved prefusion optimized envelope trimers as HIV-1 vaccine candidates. *Nature Communications* **14** (2023). https://doi.org/10.1038/s41467-023-37742-z
12. Zhang, Y.-N. *et al. Single-component self-assembling protein nanoparticles displaying stabilized prefusion-closed hemagglutinin trimers for influenza vaccine development* (Cold Spring Harbor Laboratory, 2025).
13. Powell, A. E. *et al.* A Single Immunization with Spike-Functionalized Ferritin Vaccines Elicits Neutralizing Antibody Responses against SARS-CoV-2 in Mice. *ACS Cent Sci* **7**, 183-199 (2021). https://doi.org/10.1021/acscentsci.0c01405
14. Xu, D. *et al.* Design of universal Ebola virus vaccine candidates via immunofocusing. *Proceedings of the National Academy of Sciences* **121** (2024). https://doi.org/10.1073/pnas.2316960121
15. Dickey, T. H. *et al.* Design of a stabilized RBD enables potently neutralizing SARS-CoV-2 single-component nanoparticle vaccines. *Cell Reports* **42**, 112266 (2023). https://doi.org/10.1016/j.celrep.2023.112266
16. Konrath, K. M. *et al.* Nucleic acid delivery of immune-focused SARS-CoV-2 nanoparticles drives rapid and potent immunogenicity capable of single-dose protection. *Cell Reports* **38**, 110318 (2022). https://doi.org/10.1016/j.celrep.2022.110318

17 Corbett, K. S. *et al.* Design of Nanoparticulate Group 2 Influenza Virus Hemagglutinin Stem Antigens That Activate Unmutated Ancestor B Cell Receptors of Broadly Neutralizing Antibody Lineages. *mBio* **10** (2019). https://doi.org/10.1128/mbio.02810-18

18 Kanekiyo, M. *et al.* Rational Design of an Epstein-Barr Virus Vaccine Targeting the Receptor-Binding Site. *Cell* **162**, 1090-1100 (2015). https://doi.org/10.1016/j.cell.2015.07.043

19 Yassine, H. M. *et al.* Hemagglutinin-stem nanoparticles generate heterosubtypic influenza protection. *Nature Medicine* **21**, 1065-1070 (2015). https://doi.org/10.1038/nm.3927

20 Sliepen, K. *et al.* Induction of cross-neutralizing antibodies by a permuted hepatitis C virus glycoprotein nanoparticle vaccine candidate. *Nature Communications* **13** (2022). https://doi.org/10.1038/s41467-022-34961-8

21 Chao, C. W. *et al.* Protein nanoparticle vaccines induce potent neutralizing antibody responses against MERS-CoV. *Cell Reports* **43**, 115036 (2024). https://doi.org/10.1016/j.celrep.2024.115036

22 McLeod, B. *et al.* Vaccination with a structure-based stabilized version of malarial antigen Pfs48/45 elicits ultra-potent transmission-blocking antibody responses. *Immunity* **55**, 1680-1692.e1688 (2022). https://doi.org/10.1016/j.immuni.2022.07.015

23 Malhi, H. *et al.* Immunization with a self-assembling nanoparticle vaccine displaying EBV gH/gL protects humanized mice against lethal viral challenge. *Cell Reports Medicine* **3**, 100658 (2022). https://doi.org/10.1016/j.xcrm.2022.100658

24 Sun, C. *et al.* A gB nanoparticle vaccine elicits a protective neutralizing antibody response against EBV. *Cell Host & Microbe* **31**, 1882-1897.e1810 (2023). https://doi.org/10.1016/j.chom.2023.09.011

25 He, L. *et al.* Single-component multilayered self-assembling nanoparticles presenting rationally designed glycoprotein trimers as Ebola virus vaccines. *Nature Communications* **12** (2021). https://doi.org/10.1038/s41467-021-22867-w

26 Mu, Z. *et al.* mRNA-encoded HIV-1 Env trimer ferritin nanoparticles induce monoclonal antibodies that neutralize heterologous HIV-1 isolates in mice. *Cell reports* **38**, 110514-110514 (2022). https://doi.org/10.1016/j.celrep.2022.110514
